# An exploratory trial of a single dose of CAM2043 (treprostinil subcutaneous depot) in SSc-related RP

**DOI:** 10.1093/rheumatology/keaf291

**Published:** 2025-05-29

**Authors:** Ariane L Herrick, Andrea Murray, Graham Dinsdale, Joanne Manning, Chukwuma Chukwu, Marcia Alvarez Fernandez, Ulrika Axling, James R Seibold, Fredrik Tiberg

**Affiliations:** Centre for Musculoskeletal Research, The University of Manchester, Manchester Academic Health Science Centre, Manchester, UK; Rheumatology Department, Northern Care Alliance NHS Foundation Trust, Salford, UK; Centre for Musculoskeletal Research, The University of Manchester, Manchester Academic Health Science Centre, Manchester, UK; Rheumatology Department, Northern Care Alliance NHS Foundation Trust, Salford, UK; Rheumatology Department, Northern Care Alliance NHS Foundation Trust, Salford, UK; Rheumatology Department, Northern Care Alliance NHS Foundation Trust, Salford, UK; Clinical Research Facility, Northern Care Alliance NHS Foundation Trust, Salford, UK; Clinical Development, Camurus AB, Lund, Sweden; Clinical Development, Camurus AB, Lund, Sweden; Scleroderma Research Consultants LLC, Aiken, SC, USA; Clinical Development, Camurus AB, Lund, Sweden

**Keywords:** clinical trial, extended release, FluidCrystal^®^, RP, SSc, treprostinil

## Abstract

**Objectives:**

Our aim was to explore the effect of a single subcutaneous dose of CAM2043, a novel extended-release subcutaneous formulation of treprostinil, on finger temperature in patients with SSc-related RP.

**Methods:**

This was an exploratory, open-label, single-dose Phase 2 trial. Ten female patients (median age 54.0 years) attended on six occasions: screening, baseline (day 1), days 2, 3, 8 and 15. On day 1, patients received a single subcutaneous injection of 2.5 mg CAM2043. A standard cold challenge test of the hands was performed pre-dose and at 3, 6, 24, 72, 168 and 336 h post-dose, with temperature response over the subsequent 15 min measured by infrared thermography. The primary end point was the mean change in area under the temperature-time curve (AUC_therm_) for rewarming (eight fingers) at 6 h vs baseline.

**Results:**

AUC_therm_ increased 6 h post-dose (mean increase 192.7°C×sec [95% CI: −727.1, 1112.6]) and was significantly greater at 24 h than at baseline (mean increase 1175.8°C×sec [95% CI: 127.3, 2224.3]), returning to baseline values by day 15. Maximum temperature after rewarming increased significantly from baseline at 24 h, by 1.4°C (95% CI: 0.1, 2.7). Mean (S.D.) Raynaud’s Condition Score (3.7 [1.3] units at baseline) improved significantly post-dosing, including day 8 (mean reduction 1.6 units [95% CI: −2.68, −0.52]). Adverse events were reported by all 10 patients: all reported erythema and pain at the injection site.

**Conclusion:**

The positive outcome indicates that CAM2043 could be further investigated in clinical trials of RP.

**Trial registration:**

www.clinicaltrialsregister.eu/, EudraCT number 2019–002444-24.

Rheumatology key messages

- CAM2043 is a novel extended-release subcutaneous formulation of treprostinil, a prostacyclin analogue.
- A single dose of CAM2043 was associated with increased finger rewarming and improvement in Raynaud’s.
- CAM2043 could be further investigated in clinical trials of RP.

## Introduction

Over 95% of patients with SSc experience RP with colour changes of the fingers in response to cold or emotional stress [1]. RP is one of the most distressing features of SSc with a major impact on quality of life [2, 3]: ∼50% of patients progress to digital ulceration [4]. Current treatments are often ineffective [5, 6] and alternative treatment options for SSc-related RP are an unmet clinical need. Intravenous (i.v.) administration of prostacyclin analogues is a recommended treatment [7, 8] that reduces frequency and duration of RP attacks, and also helps to heal digital ulcers [9, 10]. However, a disadvantage of this approach is the need for hospitalization, therefore IV prostanoid therapy tends to be reserved for patients with the most severe disease. Studies of oral prostanoid therapy have not provided evidence of consistent benefit [10].

Treprostinil, a prostacyclin analogue, can be administrated subcutaneously. Subcutaneous treprostinil is licenced for the treatment of pulmonary arterial hypertension, when it is given by continuous infusion. Treprostinil subcutaneous depot (CAM2043) is a novel extended-release subcutaneous formulation of treprostinil, based on the FluidCrystal^®^ technology [11, 12] and designed to produce therapeutic levels over one week.

The aim of this Phase 2 trial was to explore the effect of a single subcutaneous dose of CAM2043 on finger skin temperature in response to cold exposure (as evaluated by infrared thermography following cold challenge), in patients with SSc-related RP. Skin temperature is an indirect measure of blood flow. Other objectives were to assess the effect of CAM2043 on patient reported outcomes, plasma concentrations of treprostinil after a single subcutaneous dose of CAM2043, and safety and tolerability.

## Methods

### Study design

This was an exploratory, open-label, single-dose Phase 2 trial (EudraCT Number 2019–002444-24). Patients attended on six occasions (Supplementary Fig. S1, available at *Rheumatology* online): screening (up to 14 days before the baseline visit), baseline (day 1) and days 2, 3, 8 and 15. Patients were asked to refrain from vigorous exercise, caffeine and alcohol for 4 h prior to and during the study visits.

On day 1, all patients entered the 1-week open-label treatment period, followed by a 1-week follow-up period. After an initial standard cold challenge of the hands [13], each patient received a single subcutaneous injection of 2.5 mg of the investigational medicinal product (IMP) CAM2043, except the first patient who received 5 mg CAM2043. The cold challenge test (combined with thermographic imaging as described below) was repeated at 3, 6, 24, 72, 168 and 336 h post-dose. The 168-h (day 8) cold challenge/thermography marked the end of the 1-week treatment period and the 336 h (day 15) cold challenge/thermography the end of the 1-week follow-up period (Supplementary Fig. S1, available at *Rheumatology* online). Patients were offered prophylactic treatment with a NSAID to avoid/minimize injection site pain and swelling due to treprostinil. Other measures suggested for injection site reactions included paracetamol (or a stronger analgesic) and cold packs.

Prior to each cold challenge, patients acclimatized at 23°C for at least 20 min. The cold challenge protocol is described in full elsewhere [13]. In summary, the fingers of both hands were immersed in cold water (15°C for 1 min) to the level of the metacarpophalangeal joints. Infrared thermographic images (FLIR T540 thermal camera, FLIR Systems, Taby, Sweden) were taken immediately prior to the cold challenge, then post-cold challenge every 15 s, for 15 min, to examine rewarming.

Other assessments included the Raynaud’s Condition Score (RCS) and the Patient Global Assessment (PGA). The RCS, graded on a scale of 0 ‘no difficulty’ to 10 ‘extreme difficulty’, is a validated score of RP severity [14]. The PGA is a 5-point scale assessing whether RP was ‘much better’, ‘a little better’, ‘the same’, ‘a little worse’ or ‘much worse’. RCS and PGA were assessed daily using paper diaries.

Pharmacokinetic (PK) samples for plasma treprostinil concentration determination were collected pre-dose and at ∼3, 6, 24, 72, 168 and 336 h.

The study was approved by the North West—Greater Manchester Central Research Ethics Committee. All patients signed written informed consent.

### Outcome measures

The primary end point was the mean change from baseline at 6 h post-dose in the area under the temperature–time curve (AUC_therm_) for rewarming (eight fingers), adjusted for the pre-dose, pre-cold challenge temperature and measured in units of °C × sec. This adjusted value is usually negative as the fingers of patients with SSc seldom rewarm fully within a 15-min period. Secondary endpoints included: change from baseline in AUC_therm_ at other time-points, maximum temperature after rewarming (MAX), plasma treprostinil levels, RCS and PGA. Safety and tolerability were assessed throughout the trial. Assessment of safety included recording of adverse events (AEs) and vital signs at each visit, and at screening and day 15, laboratory assessments (haematology and clinical chemistry) and electrocardiogram. The tolerability assessments of erythema and swelling at the injection site were each assessed by the investigator on a 0–3-point rating (0 = none, 1 = mild, 2 = moderate, 3 = severe), and pain was rated by patients on a 0–10 numerical rating scale (0 = no pain, 10 = worst possible pain).

### Patients

Patients were eligible for recruitment if they had a diagnosis of SSc as defined by the 2013 ACR/EULAR criteria for SSc [15], were aged ≥18 years and ≤65 years, and experienced at least five attacks of RP per week. Exclusion criteria included the presence of active digital ulceration within the 4 weeks prior to screening, use of tobacco-related products within 6 months of screening, history of any clinically significant allergic conditions, pregnancy and breast-feeding. Patients were allowed to continue on stable vasodilator therapy, for example calcium challenge blockers and phosphodiesterase inhibitors, throughout the trial (no dose adjustments for at least 4 weeks prior to the screening visit). However, parenteral, inhaled or oral prostacyclin or prostacyclin receptor agonists were not allowed within 12 weeks of screening and during the trial.

### Statistical analysis

Primary and secondary efficacy endpoints were analysed using descriptive statistics, including 95% CIs. Thermographic parameters were summarized across the eight fingers by averaging. Pharmacokinetic and safety analyses were also reported descriptively. For missing data, the imputation method of last observation carried forward (LOCF) was applied. As this was an exploratory trial, no formal sample size calculation was made. It was assumed that inclusion of 12 patients would provide sufficient data on levels and variability of the measurements to inform future trials.

## Results

The study was conducted between 19 November 2020 and 14 December 2021. A total of 11 patients were screened, one of whom did not fulfil the eligibility criteria (Supplementary Fig. S2, available at *Rheumatology* online). All 10 patients were female, median age 54.0 years (range 43.0–65.0), all had the limited cutaneous subtype of SSc, and all were anticentromere antibody positive. Median (range) SSc duration was 7.0 (1.0–27.0) years and RP duration 14.5 (5.0–32.0) years (Supplementary Table S1, available at *Rheumatology* online). Six patients were on a calcium channel blocker, four an angiotensin receptor blocker and one a phosphodiesterase inhibitor for treatment of RP. All 10 patients receiving IMP completed the study, with results analysed for safety and efficacy.

### Thermographic parameters

**Figure 1.**
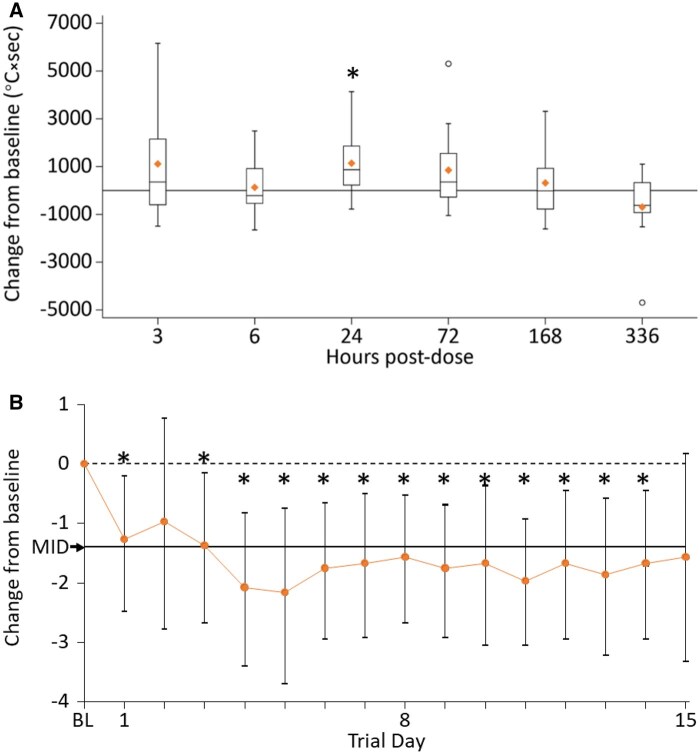
Mean change from baseline in (**A**) AUC_therm_ and (**B**) RCS over the 15-day study period. (**A**) All AUC_therm_ measurements (in °C×sec) were averaged across eight fingers. Boxes show quartile range (QR), diamonds mark mean values, in-box lines mark median values, whiskers range values <1.5 × QR outside of QR, circles mark outliers. (**B**) BL indicates baseline, that is, the last non-missing RCS value obtained before administration of CAM2043 on day 1. Asterisks indicate statistically significant differences as 95% CI excludes zero. AUC_therm_: area under the curve for perfusion, adjusted for pre-dose, pre-cold challenge temperature, as derived from thermography measurements; BL: baseline; MID: minimally important difference; RCS: Raynaud’s Condition Score

Table 1 presents the thermographic parameters by time point. AUC_therm_ increased 6 h post-dose compared with baseline, but not statistically significantly: mean increase 192.7°C×sec (95% CI: −727.1, 1112.6). AUC_therm_ was significantly greater at 24 h than at baseline (mean increase 1175.8°C×sec [95% CI: 127.3, 2224.3]) with a trend towards mean AUC_therm_ values higher than baseline up to 168 h post-dose (day 8) and returning to baseline values at 336 h (day 15) (Fig. 1A). A statistically significant increase from baseline was also seen for MAX at 24 h: mean increase 1.4°C (95% CI: 0.1, 2.7). At other time-points MAX was not significantly different from baseline. The results for the remaining thermography endpoints (distal–dorsal temperature difference, pre-cold challenge temperature and rewarming gradient during first 2 min) showed trends similar to that observed for AUC_therm_ but no values were significantly different from baseline at any of the time-points measured (Table 1).

**Table 1.** Thermographic parameters, plasma levels of treprostinil and patient reported outcomes in 10 patients with SSc at the different study time-points

Time-point (trial day)	1			2	4	8	15
**Thermographic parameters^a^**	**Pre-dose^b^**	**3 h**	**6 h**	**24 h**	**72 h**	**168 h**	**336 h**
*n*	10	10	10^c^	10^c^	10	10	10^c^
Pre-cold challenge temperature (°C) Mean (S.D.)	28.0 (2.6)	28.7 (3.4)	28.5 (2.4)	28.7 (3.6)	28.7 (2.9)	28.6 (2.7)	27.7 (3.0)
AUC _therm_ (°C×sec) Mean (S.D.)	−4118.4 (1132.1)	−2965.5 (2494.8)	−3925.6^d^ (2182.6)	−2942.6 (2384.1)	−3214.5 (2444.0)	−3740.4 (2240.8)	−4766.8 (1677.2)
MAX (°C) Mean (S.D.)	24.6 (3.5)	26.5 (4.9)	25.0 (3.7)	26.0 (4.9)	25.9 (4.4)	25.4 (3.8)	23.8 (3.3)
Gradient (°C/min) Mean (S.D.)	0.7 (0.2)	0.9 (0.5)	0.8 (0.2)	0.9 (0.7)	0.9 (0.3)	0.7 (0.4)	0.6 (0.2)
Distal–dorsal difference (°C) Mean (S.D.)	−2.4 (1.7)	−1.8 (1.6)	−2.0 (1.5)	−2.1 (2.2)	−2.1 (2.1)	−1.9 (1.5)	−2.5 (1.7)
**Plasma levels of treprostinil (ng/ml)^e^**	**Pre-dose^b^**	**3 h**	**6 h**	**24 h**	**72 h**	**168 h**	**336 h**
*n*	10	10	9	8	10	10	10
Mean (S.D.)	0.0000 (0.0000)	1.5615 (0.6937)	2.1478 (1.0366)	0.5700 (0.2616)	0.3252 (0.1897)	0.1182 (0.0645)	0.0474 (0.0432)
**Patient reported outcomes^e^**	**Day 1 pre-dose^b^**	**Day 1 post-dose**		**Day 2**	**Day 4**	**Day 8**	**Day 15**
*n*	10	9		8	9	10	7
Raynaud’s condition score Mean (S.D.)	3.7 (1.3)	2.2 (1.4)		2.6 (1.2)	1.9 (1.4)	2.1 (1.2)	2.1 (1.8)
PGA; *n* (%)							
Much better	N/A	1 (10.0)		2 (20.0)	2 (20.0)	0	1 (10.0)
A little better	N/A	2 (20.0)		2 (20.0)	4 (40.0)	4 (40.0)	3 (30.0)
The same	N/A	6 (60.0)		3 (30.0)	3 (30.0)	6 (60.0)	1 (10.0)
A little worse	N/A	0		1 (10.0)	0	0	2 (20.0)
Much worse	N/A	0		0	0	0	0

AUC_therm_: the area under the temperature–time curve for rewarming adjusted for the pre-dose, pre-cold challenge temperature, as derived from thermography measurements (units = °C × seconds); Distal–dorsal difference: temperature difference between the dorsum of the hand and fingertip; Gradient: measured value of rewarming curve gradient in first 2 min post-cold challenge; MAX: maximum temperature post-cold challenge (mean across all eight fingers); *n*: number of patients included in the analysis; N/A: not applicable; PGA: Patient Global Assessment.

The full analysis set was defined as all patients who were administered CAM2043 and who had data from at least one time point after dosing.

a Thermographic parameters were averaged across all eight fingers to give a mean value for each participant, based on last observation carried forward (LOCF) full analysis set.

b Pre-dose measurement on day 1 was baseline.

c The actual number of patients who provided thermographic parameters was nine patients at the 6-h assessment, eight patients at the 24-h assessment and nine patients at the 336-h assessment.

d Primary end point.

e Based on the full analysis set.

### Raynaud’s Condition Score

At baseline, mean (S.D.) RCS was 3.7 (1.3) units (10-point scale), and was significantly improved post-dosing, with the greatest improvements at 4 days and 5 days post-dose, with a mean RCS reduction of 2.1 units (95% CI: −3.41, −0.81) on day 4, 2.2 units (95% CI: −3.70, −0.74) on day 5 and 1.6 units (95% CI: −2.68, −0.52) on day 8. These improvements were statistically significant over the entire 15-day post-dose period, except on day 2 and day 15 (Fig. 1B) and are greater than the minimally important difference of 1.4–1.5 [16].

### Patient Global Assessment

At the end of the treatment period (day 8), four patients (40%) reported being ‘a little better’, and six patients (60%) ‘the same’. On day 15, one patient (10%) reported being ‘much better’, three patients ‘a little better’, one patient ‘the same’ and two patients ‘a little worse’ (assessment missing for three patients).

### Plasma treprostinil levels

The mean (S.D.) concentration (observed across nine patients) was highest 6 h post-dose at 2.1478 (1.0366) ng/ml and slowly declined over time to close to baseline levels at 336 h post-dose (0.0474 [0.0432] ng/ml). There was a delay of up to 1 day in thermography response following peak plasma treprostinil concentration (data not shown).

### Adverse events

There were no serious AEs and no dropouts due to AEs. Treatment emergent AEs (TEAEs) were reported in all 10 (100.0%) patients and all 10 patients reported at least one injection site-related TEAE (erythema and pain in all 10 patients; swelling in nine patients) (Supplementary Table S2, available at *Rheumatology* online, for details on other AEs). In total, 127 TEAEs were reported, of which 115 were assessed as related to treatment (77 injection site-related). Four (40.0%) patients experienced severe CAM2043-related TEAEs (all transient). Five patients (50%) commenced an analgesic during the trial and five (50%) an NSAID because of injection site problems, which are well known reactions to treprostinil. No clinically meaningful changes were noted in any of the patients in laboratory values (haematology, coagulation, biochemistry), vital signs or ECG. Local tolerability assessments peaked on day 4: clinician-assessed erythema ‘severe’ in one patient (10%), ‘moderate’ in eight (80%), ‘mild’ in one (10%); clinician-assessed swelling severe in three (30%), ‘moderate’ in five (50%), ‘mild’ in one (10%) and none in one (10%); and patient-assessed mean (S.D.) pain at the injection site (numeric scale 0–10) of 5.0 (2.6). By day 8 mean (S.D.) pain at the injection site had reduced to 2.3 (1.5).

## Discussion

The main findings of this exploratory Phase 2 trial were that finger skin temperature rose after CAM2043 treatment (significantly at 24 h post-dose), with significant improvements in RP symptoms (as measured by RCS) over the 15-day period following dosing, indicating a positive treatment effect. Overall, the safety profile for CAM2043 was consistent with known safety profile for treprostinil, including injection site reactions. The small number of patients studied, and the heterogeneity across patients in the response to cold challenge, have contributed to wide CIs.

It had been anticipated that the maximal effect of CAM2043 on reperfusion (as assessed by rewarming) would be at 6 h (hence the choice of primary end point). The results from the present trial in RP patients show that perfusion response appears to be delayed (maximal at 24 h), indicating a lag-phase between peak plasma exposure and digital vascular perfusion, and this needs to be taken into account in future trials.

The main limitation to future development of CAM2043 is the propensity for injection site reactions, reflected by the relatively high number of injection site-related TEAEs observed during local tolerability assessments in the trial. These reactions are expected as treprostinil is well recognized to be locally irritating when administered subcutaneously or intravenously [17–19]. NSAIDs or other analgesics can limit injection site reactions. However, NSAIDs can be associated with adverse gastrointestinal effects, and so need to be used with some caution in patients with SSc.

Limitations of the study were the small number of patients studied (including that all were female and all white, limiting the generalizability of the results) and the open-label design in a single-dose setting. This means that results should be interpreted with caution. However, its design was appropriate for an exploratory study, and as such it has provided valuable insight into a potential new mode of therapy for SSc-related RP which could be administered by patients themselves, in their own homes. The fact that all patients were anticentromere positive suggests that those who agreed to participate were those with significant digital vasculopathy [20], who recognized the need for alternatives to i.v. prostanoid therapy.

The study has also further confirmed feasibility of using thermographic response to a cold challenge as an outcome measure in Phase 2 trials. In conclusion, CAM2043 could be further investigated in comparator-controlled clinical trials of RP in patients of both sexes, different races and ethnicities, and with different disease severities. Future challenges include identifying the optimal formulation of CAM2043 to balance therapeutic and adverse effects.

## Supplementary Material

keaf291_Supplementary_Data

## Data Availability

Restrictions apply to the availability of some or all data generated or analysed during this study to preserve patient confidentiality. The corresponding author will on request detail the restrictions and any conditions under which access to some data may be provided.
