# Supplementary material for: An exploratory trial of a single dose of CAM2043 (treprostinil subcutaneous depot) in SSc-related RP
Source: Rheumatology (Oxford). 2025 May 29;64(10):5498–503. doi: 10.1093/rheumatology/keaf291 (PMC12494207; doi:10.1093/rheumatology/keaf291)
Supplement: keaf291_Supplementary_Data [file keaf291_supplementary_data.docx]

SUPPLEMENTARY MATERIAL - An Exploratory Trial of a Single Dose of CAM2043 (Treprostinil Subcutaneous Depot) in Systemic Sclerosis-related Raynaud’s Phenomenon

**Supplementary Figure S1: Schematic design of the trial**

**
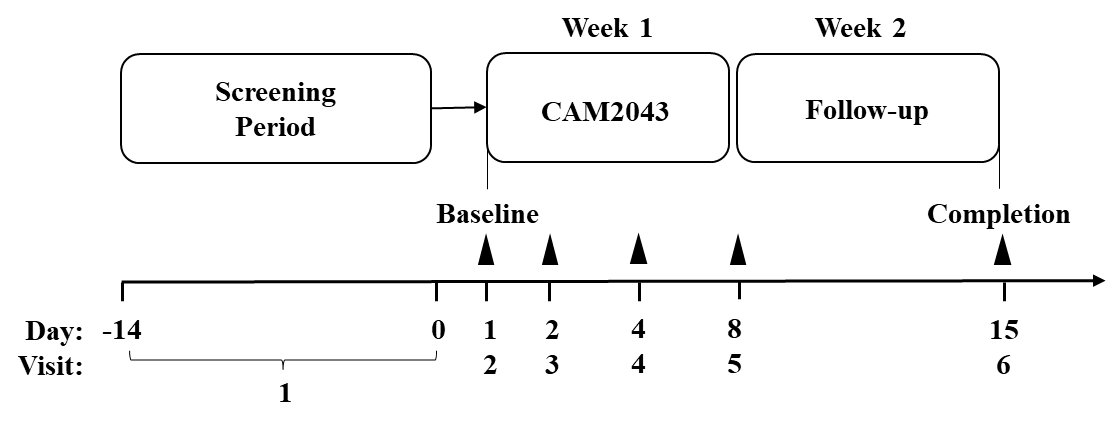
**

Triangles indicate days when cold challenges were performed. On Day 1, cold challenges were performed pre-dose and at 3 and 6 hours post-dose.

**Supplementary Figure S2: Disposition of patients**

**
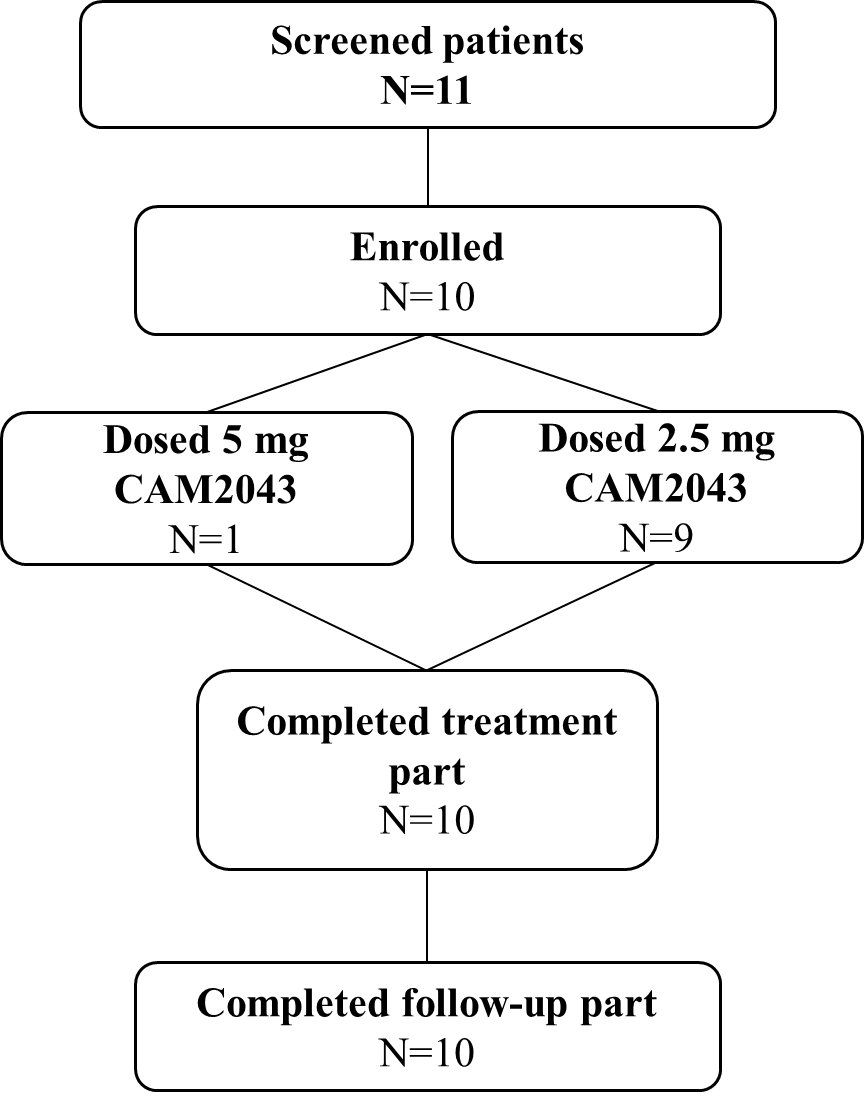
**

The patient who received the 5-mg CAM2043 dose presented with 5 severe events (injection site erythema/pain/swelling, headache, pain in jaw). For this reason, the protocol was amended and the dose reduced to 2.5 mg for the remaining patients.

**Supplementary Table S1: Demographics and baseline characteristics of the 10 patients receiving trial treatment**

| **Number of patients** | 10 |
| --- | --- |
| **Age** (years) | 54.0 (43.0; 65.0) |
| **Sex** (females); N (%) | 10 (100) |
| **Race** (white); N (%) | 10 (100) |
| **Weight** (kg) | 69.4 (55.8;90.2) |
| **Body mass index (BMI)** (kg/m^2^) | 24.3 (20.5;33.5) |
| **Duration of Raynaud's phenomenon** (years) | 14.5 (5.0;32.0) |
| **Duration of SSc** (years) | 7.0 (1.0;27.0) |
| **Limited cutaneous SSc subtype**; N (%) | 10 (100) |
| **Anticentromere antibody positive:** N (%) | 10 (100) |
| **Raynaud Condition Score** (mean [SD]) | 3.7 (1.3) |

Values are Median (range) unless otherwise specified

**Supplementary Table S2: TEAEs assessed as related to CAM2043 (other than injection site-related TEAEs) and reported in more than one patient**

| TEAE | Number (%) of patients |
| --- | --- |
| Headache | 8 (80) |
| Diarrhoea | 5 (50) |
| Vomiting | 4 (40) |
| Flushing | 4 (40) |
| Nausea | 2 (20) |
| Pain in jaw | 2 (20) |
| Sinus headache | 2 (20) |
